# Association of kidney function-related dietary pattern, weight status, and cardiovascular risk factors with severity of impaired kidney function in middle-aged and older adults with chronic kidney disease: a cross-sectional population study

**DOI:** 10.1186/s12937-019-0452-4

**Published:** 2019-04-22

**Authors:** Adi Lukas Kurniawan, Chien-Yeh Hsu, Hsiao-Hsien Rau, Li-Yin Lin, Jane C.-J. Chao

**Affiliations:** 1 School of Nutrition and Health Sciences, College of Nutrition, Taipei Medical University, 250 Wu-Hsing Street, Taipei, 110 Taiwan; 2 Department of Information Management, National Taipei University of Nursing and Health Sciences, 365 Ming-Te Road, Peitou District, Taipei, 112 Taiwan; 3 Master Program in Global Health and Development, College of Public Health, Taipei Medical University, 250 Wu-Hsing Street, Taipei, 110 Taiwan; 4 Joint Commission of Taiwan. 31 Sec.2 Sanmin Road, Banqiao District, New Taipei City, 220 Taiwan; 5 Nutrition Research Center, Taipei Medical University Hospital, 252 Wu-Hsing Street, Taipei, 110 Taiwan

**Keywords:** Dietary pattern, Reduced rank regression, Weight status, Cardiovascular risk factors, Kidney function

## Abstract

**Background:**

Chronic Kidney Disease (CKD), characterized by impaired kidney function, affects over 1.5 million individuals in Taiwan. Cardiovascular disease (CVD) is commonly found in patients with CKD, and the increased prevalence of obesity can have some implications for the risk of both CKD and CVD. Since diet plays an important role in the development of obesity, CVD and CKD, our study was designed to investigate the association of kidney function-related dietary pattern with weight status, cardiovascular risk factors, and the severity of impaired kidney function in middle-aged and older adults in Taiwan.

**Methods:**

A total of 41,128 participants aged 40 to 95 years old with an estimated glomerular filtration rate (eGFR) less than 90 mL/min/1.73 m^2^ and proteinuria were recruited from Mei Jau Health Institute between 2008 and 2010. The kidney function-related dietary pattern was identified using reduced rank regression (RRR) and was known as high consumption of preserved or processed food, meat, organ meats, rice/flour products, and, low consumption of fruit, dark-colored vegetables, bread, and beans. A multivariable logistic regression analysis was used to identify the association of weight status and cardiovascular risk factors with moderately/severely impaired kidney function (eGFR < 60 mL/min/1.73 m^2^) and the association of dietary pattern with the outcomes aforementioned.

**Results:**

Moderately/severely impaired kidney function participants were heavier and had higher abnormality of cardiovascular risk factors compared with those with mildly impaired kidney function. Weight status (OR = 1.28, 95% CI 1.12–1.45, *P* <  0.001 for obesity) and cardiovascular risk factors (OR = 1.52, 95% CI 1.31–1.77, *P* <  0.001 for high total cholesterol/HDL-C ratio and OR = 1.56, 95% CI 1.41–1.72, *P* <  0.001 for hypercalcemia) were positively associated with increased risk of moderately/severely impaired kidney function. The kidney function-related dietary pattern was correlated with overweight or obese (OR = 2.07, 95% CI 1.89–2.27, *P* <  0.01) weight status, increased cardiovascular risk by 10–31%, and the risk of moderately/severely impaired kidney function (OR = 1.15, 95% CI 1.02–1.29, *P* <  0.05).

**Conclusions:**

The RRR-derived kidney function-related dietary pattern, characterized by high intake of processed and animal foods and low intake of plant foods, predicts the risks for developing cardiovascular disease and moderately/severely impaired kidney function among middle-aged and older adults.

**Electronic supplementary material:**

The online version of this article (10.1186/s12937-019-0452-4) contains supplementary material, which is available to authorized users.

## Introduction

Chronic kidney disease (CKD), characterized by impaired kidney function, has surfaced as a global health problem. In Taiwan, the prevalence of CKD stage 1–5 in 2007 was 9.8–11.9%, meaning more than 1.5 million individuals suffered from CKD [1]. Cardiovascular disease (CVD) is an adverse outcome of kidney disease and is associated with increased major causes of mortality and morbidity [2]. A population-based prospective cohort study in Iceland reported that adjusted hazard ratio (HR) for CVD was 1.55 to 4.29 in CKD stage 1 to 4 [3], and thus CKD was associated with increased risk for CVD mortality by 100% (HR = 2.00, 95% CI 1.78–2.25) [4]. The traditional risk factors for CVD including hypertension, diabetes, lipid abnormalities, and obesity have been known as important determinants for the risk of developing CVD in patients with CKD [5, 6]. Moreover, abnormal calcium and phosphorus metabolism represented as non-traditional CVD risk factors. Both high calcium and phosphorus levels can directly increase vascular calcification [7]. This alteration in mineral metabolism characterized by hypercalcemia and elevated serum phosphate levels are common in patients with CKD and may lead to calcification and other cardiovascular events [8]. However, few studies in Taiwan have investigated whether abnormal weight status and both traditional as well as non-traditional CVD risk factors are associated with the severity of impaired kidney function.

In addition, diet also have been associated with cardiovascular risk factors and other health-related outcomes. A healthy dietary pattern, characterized by high consumption of whole grains, fruit, vegetables, and unsaturated oil, was correlated with reduced cardiovascular risk factors and increased kidney function [9, 10]. In contrast, a western dietary pattern with high consumption of deep-fried foods, processed foods, meat, and organ meats was positively associated with increased cardiovascular risk factors and progression of CKD [11, 12]. In this study, we used reduced rank regression (RRR) [13] to derive kidney function-related dietary pattern. The RRR method is a multivariable linear functions where it combines a priori and a posteriori approaches to derive dietary patterns [14]. Recently, RRR has been widely used to assess dietary patterns in several studies [15–17]. By using this method, researchers are able to identify a linear combination of predictor variables, select response variables based on prior knowledge, and find dietary patterns related to the disease of interest [13, 14]. Food items or food groups derived from food frequency questionnaire (FFQ) have been used as predictor variables, while response variables refer to nutrients or blood biomarkers as early predictors of a disease [13, 14]. Additionally, compared with other method for deriving dietary patterns such as principal component analysis (PCA), the RRR method is more likely to be associated with health-related outcomes [13, 18, 19]. To our knowledge, there is no study using RRR to derive dietary patterns that are associated with kidney function. Therefore, the aims of this study were to (1) investigate the association of abnormal weight status and cardiovascular risk factors with the severity of impaired kidney function and (2) identify whether RRR-derived kidney function-related dietary pattern is associated with abnormal weight status, cardiovascular risk factors, and the severity of impaired kidney function among middle-aged and older participants with CKD.

## Methods

### Study participants

This study was conducted using health-screening data from Mei Jau (MJ) Health Institute, Taiwan. The MJ Health Institute is a private institute with four health-screening centers (Taipei, Taoyuan, Taichung, and Kaohsiung) in Taiwan, and it provides periodic health check-up (on average one examination per year per person) to its members. All participants had a series of health check-up including anthropometric assessment, blood tests, stool and urine tests, physical examination, and completed a self-reported questionnaire to collect information about sociodemographic, lifestyle, medical history as well as dietary habits. In addition, every participant had signed the consent form authorized by the MJ Health Institute for research purpose only and no personal identification information would be released. The Joint Institutional Review Board of Taipei Medical University (TMU-JIRB N201802006) approved this study.

### Data collection

We included 151,206 participants with an estimated glomerular filtration rate (eGFR) less than 90 mL/min/1.73 m^2^ and proteinuria from the MJ Health Institute database between years of 2008 and 2010. After excluding 110,078 participants who were (1) aged less than 40 y (*n* = 39,066), (2) with any disease condition such as cancer, cirrhosis, autoimmune disease, or virus infection (*n* = 48,169), (3) with history of kidney surgery (*n* = 1765), (4) with error results in blood analysis (*n* = 1128), (5) failed to complete the questionnaire (*n* = 212), (6) with missing data in dietary habit (*n* = 11,184), or (7) with multiple entries in the database (*n* = 8554), a total of 41,128 participants were included in this study.

### Clinical and biochemical data and definition of the disease

Body weight, height, waist or hip circumference, body fat mass, and blood pressure were measured by an auto-anthropometers during health check-up. Fasting blood glucose (FBG), triglycerides (TG), total cholesterol (TC), high density lipoprotein-cholesterol (HDL-C), low density lipoprotein-cholesterol (LDL-C), C-reactive protein (CRP), blood urea nitrogen (BUN), creatinine, albumin, calcium, and phosphorus were analyzed at the MJ Health Institute’s central laboratory. Body Mass Index (BMI) status was defined as follows: normal (18.5 kg/m^2^ ≤ BMI < 24 kg/m^2^), overweight (24 kg/m^2^ ≤ BMI < 27 kg/m^2^), and obese (BMI ≥27 kg/m^2^) [20]. High waist circumference was defined as waist circumference ≥ 80 cm for female and ≥ 90 cm for male [21]. High waist-to-hip ratio (WHR) was defined as ≥0.85 for female and ≥ 0.90 for male [21]. High body fat mass was defined as body fat mass ≥ 35% for female and ≥ 24% for male [22]. Hypertension was defined as having at least one of the followings: (1) systolic blood pressure (SBP) ≥ 140 mmHg, (2) diastolic blood pressure (DBP) ≥ 90 mmHg, (3) use of antihypertensive medication, or (4) self-reported hypertension [23]. Diabetes was defined as at least one of the followings: (1) FBG ≥ 7.0 mmol/L (≥ 126 mg/dL), (2) use of hypoglycemic medication, or (3) self-reported diabetes [24]. The definition of abnormal blood lipids were TG ≥ 2.3 mmol/L (≥ 200 mg/dL) for high TG, TC ≥ 6.2 mmol/L (≥ 240 mg/dL) and/or use of lipid-lowering drugs for high TC, HDL-C < 1.0 mmol/L (< 40 mg/dL) for low HDL-C, LDL-C ≥ 4.1 mmol/L (≥ 160 mg/dL) and/or use of lipid-lowering drugs for high LDL-C [25], and TC-to-HDL-C ratio (TC/HDL-C ratio) ≥ 5.0 for high TC/HDL-C ratio [26].

Proteinuria was reported as one or more pluses (+). We used the Modification of Diet in Renal Disease Study (MDRD) equation to calculate eGFR as an indicator of kidney function [27]:

$$ \mathrm{eGFR}=186.3\times {\left(\mathrm{serum}\ \mathrm{creatinine}\ \mathrm{in}\ \mathrm{mg}/\mathrm{dL}\right)}^{\hbox{-} 1.154}\times {\left(\mathrm{age}\right)}^{\hbox{-} 0.203}\times \left(0.742\ \mathrm{if}\ \mathrm{female}\right) $$

Moreover, based on eGFR levels, we further classified impaired kidney function into two categories: (1) mildly impaired (stage 2) defined as eGFR at 60–89 mL/min/1.73 m^2^ and (2) moderately/severely impaired (stage 3–5) defined as eGFR < 60 mL/min/1.73 m^2^ [28]. Hypercalcemia was defined as serum calcium levels ≥2.37 mmol/L based on National Kidney Foundation guidelines [29], while serum phosphorus levels were categorized into high (≥ median) or low (< median) level.

### Dietary assessment and other covariates

Dietary assessment was evaluated using a standardized and validated self-administered semi-quantitative food frequency questionnaire (SQ-FFQ) [11, 30]. The frequency and servings of dietary intake were investigated according to the consumption of twenty-two food groups at per day or per week in the past month and categorized into five response options as previously described [11]. The other covariates collected using a self-reported questionnaire were age, smoking status (none, former, and current), drinking status (no: < 1 time/week or yes: ≥ 1–2 times/week), physical activity status (no: < 1 h/week or yes: ≥ 1–2 h/week), medical history of CVD, hypertension, or diabetes, and use of cardiovascular, hypertension, or diabetes medication. High cardiovascular risk profile was defined as having a history of CVD and/or use of cardiovascular medication. However, participants who had been previously diagnosed with CVD might have changed their lifestyle, and thus we decided to adjust for CVD risk profile in the analysis.

### Statistical analysis

Statistical analysis was performed by using SAS 9.4 (SAS Institute Inc., Cary, NC, USA) and IBM SPSS 20 (IBM Corp., Armonk, NY, USA). Continuous and categorical variables are presented as a mean ± standard deviation (SD) and a number (percentage), respectively. A Mann-Whitney *U* test and a chi-square test were used for comparing the baseline characteristics between two continuous and categorical groups, respectively. A multivariable logistic regression analysis, expressed as odds ratios (OR) and 95% confidence intervals (CIs), was performed to identify: (1) the association between weight status and cardiovascular risk factors with moderately/severely impaired kidney function and (2) the association between dietary pattern scores across tertiles with weight status, cardiovascular risk factors, and the severity of impaired kidney function. A *P*-value < 0.05 was considered statistically significant.

**Fig. 1:**
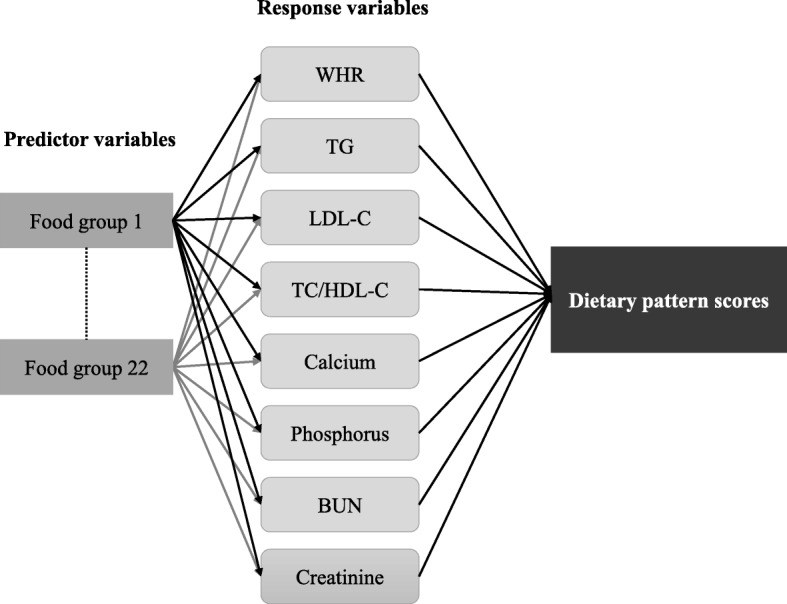
The dietary pattern derived from the reduced rank regression model. WHR waist-to-hip ratio, TG triglycerides, LDL-C low density lipoprotein-cholesterol, TC/HDL-C total cholesterol-to-high density lipoprotein-cholesterol ratio, BUN blood urea nitrogen

Dietary pattern associated with kidney function was identified by RRR using PROC PLS function in SAS 9.4. In the RRR, food groups and biomarkers were used as predictor and response variables, respectively (Fig. 1). The RRR method focuses on identifying linear functions of food groups, which explained as much variation as possible in a set of intermediate response variables [14]. We identified the response variables based on the significant correlation between eGFR and other variables by using Spearman’s correlation after adjustment with age, gender, BMI, smoking status, drinking status, physical activity, high cardiovascular risk profile, hypertension status, diabetes status, albumin, and CRP (Additional file 1: Table S1). The absolute value of factor loading ≥0.20 were selected to derive dietary pattern associated with kidney function. When eight response variables identified by Spearman’s correlation were included in the RRR, eight dietary factors were derived. Finally, we retained only the first dietary factor for the analysis as it explained the largest amount of variation in response variables. Moreover, RRR allows researchers to identify the percentage of explained variation in each food group corresponding to the response variables. This explained variation would contribute to the factor loading in each food group, meaning that food groups with the greater explained variation will produce greater factor loading.

## Results

### Characteristics of the participants

In our study, 37,882 (92.1%) middle-aged and older participants had mildly impaired kidney function and 3246 (7.9%) participants had moderately to severely impaired kidney function. The mean age and eGFR levels were 52.6 ± 9.9 y and 73.7 ± 9.9 mL/min/1.73 m^2^, respectively. The prevalence rate of overweight, obesity, high waist circumference, high WHR, and high body fat mass were 30.2, 16.7, 24.8, 38.1, and 37.0% respectively (data not shown). The prevalence rate of hypertension, diabetes, high TG, high TC, low HDL-C, high LDL-C, high TC/HDL-C ratio, and hypercalcemia was 28.6, 9.7, 13.7, 18.3, 5.8, 14.2, 8.8, and 36.8%, respectively (data not shown). Moreover, participants with moderately/severely impaired kidney function were older, heavier, had higher blood pressure, blood glucose, blood lipids, and CRP levels, but lower albumin levels compared with those who had mildly impaired kidney function (Table 1).

**Table 1:** Characteristics of the participants aged ≥40 years old by kidney function status obtained from MJ Health Institute between 2008 and 2010

	Total (*n* = 41,128)	Mildly impaired kidney function^a^ (*n* = 37,882)	Moderately/Severely impaired kidney function^b^ (*n* = 3246)	*P* ^c^
Age (y)	52.6 ± 9.9	51.7 ± 9.3	62.9 ± 11.1	< 0.001
Sex, males	21,376 (52.0)	19,682 (52.0)	1694 (52.2)	0.800
Smoking status, current	6717 (16.3)	6304 (16.6)	413 (12.7)	< 0.001
Drinking status, yes	6420 (15.6)	6032 (15.9)	388 (12.0)	< 0.001
Physical activity, yes	10,814 (26.3)	9988 (26.4)	816 (25.1)	< 0.001
High cardiovascular risk profile	2489 (6.1)	1933 (5.1)	556 (17.1)	< 0.001
Hypertension status	11,779 (28.6)	10,008 (26.4)	1771 (54.6)	< 0.001
Diabetes status	3974 (9.7)	3291 (8.7)	683 (21.0)	< 0.001
Weight status				
BMI (kg/m^2^)	24.0 ± 3.3	23.9 ± 3.3	24.7 ± 3.5	< 0.001
Waist circumference	79.7 ± 9.7	79.4 ± 9.7	82.5 ± 10.1	< 0.001
Hip circumference	95.2 ± 5.9	95.2 ± 5.9	95.4 ± 6.1	0.044
WHR	0.9 ± 1.4	0.9 ± 1.4	0.9 ± 0.1	< 0.001
Body fat mass (%)	27.4 ± 6.9	27.3 ± 6.8	27.9 ± 7.5	< 0.001
Cardiovascular risk factors				
SBP (mmHg)	122.5 ± 18.2	121.8 ± 17.8	131.0 ± 20.2	< 0.001
DBP (mmHg)	73.8 ± 11.8	73.6 ± 11.7	76.2 ± 12.1	< 0.001
FBG (mmol/L)	5.9 ± 1.3	5.8 ± 1.2	6.3 ± 1.9	< 0.001
TG (mmol/L)	1.4 ± 1.1	1.4 ± 1.0	1.6 ± 1.2	< 0.001
TC (mmol/L)	5.3 ± 0.9	5.3 ± 0.9	5.4 ± 1.0	< 0.001
HDL-C (mmol/L)	1.5 ± 0.4	1.5 ± 0.4	1.5 ± 0.4	< 0.001
LDL-C (mmol/L)	3.1 ± 0.8	3.1 ± 0.8	3.2 ± 0.8	< 0.001
TC/HDL-C	3.6 ± 0.9	3.6 ± 0.9	3.8 ± 1.0	< 0.001
Calcium (mmol/L)	2.3 ± 0.1	2.3 ± 0.1	2.4 ± 0.1	< 0.001
Phosphorus (mmol/L)	1.1 ± 0.2	1.1 ± 0.1	1.2 ± 0.2	0.816
Albumin, inflammatory biomarker, and kidney function				
Albumin (g/dL)	4.5 ± 0.2	4.5 ± 0.2	4.4 ± 0.2	< 0.001
CRP (nmol/L)	22.5 ± 47.9	21.4 ± 43.4	33.7 ± 81.0	< 0.001
BUN (mmol/L)	5.2 ± 1.5	5.0 ± 1.2	6.9 ± 2.8	< 0.001
Creatinine (μmol/L)	89.5 ± 22.2	86.8 ± 13.7	120.5 ± 54.8	< 0.001
eGFR (mL/min/1.73 m^2^)	73.7 ± 9.9	75.7 ± 7.5	52.6 ± 8.6	< 0.001
Proteinuria				< 0.001
+ 1	39,311 (95.6)	36,623 (96.7)	2688 (82.8)	
+ 2	1041 (2.5)	819 (2.2)	222 (6.8)	
≥ + 3	776 (1.9)	440 (1.1)	336 (10.4)	

BMI body mass index, WHR waist-to-hip ratio, SBP systolic blood pressure, DBP diastolic blood pressure, FBG fasting blood glucose, TG triglycerides, TC total cholesterol, HDL-C high density lipoprotein-cholesterol, LDL-C low density lipoprotein-cholesterol, TC/HDL-C total cholesterol-to-HDL-C ratio, CRP C-reactive protein, BUN blood urea nitrogen, eGFR estimated glomerular filtration rate. Continuous data are presented as mean ± SD, and categorical data are presented as numbers (percentage)

^a^Mildly impaired kidney function was defined as eGFR 60–89 mL/min/1.73 m^2^

^b^Moderately/severely impaired kidney function was defined as eGFR < 60 mL/min/1.73 m^2^

^c^The *P*-value was analyzed using Mann-Whitney *U* test for continuous variables and chi-square test for categorical variables

### Weight status and cardiovascular risk factors in relation to the severity of impaired kidney function

We next investigated the association of weight status and cardiovascular risk factors with the severity of impaired kidney function (Table 2). A fully-adjusted multivariable logistic regression analysis (model 2) showed that participants who were overweight, obese, or had high WHR were significantly associated with a higher risk of moderately/severely impaired kidney function (overweight: OR = 1.25, 95% CI 1.12–1.39, *P* <  0.001, obesity: OR = 1.28, 95% CI 1.12–1.45, *P* <  0.001, and high WHR: OR = 1.11, 95% CI 1.00–1.23, *P* = 0.039, respectively) compared with normal weight participants. Meanwhile, high waist circumference only showed the tendency to be associated with the severity of impaired kidney function (*P* = 0.052). Cardiovascular risk factors were positively associated with moderately/severely impaired kidney function (*P* <  0.001), and high TC/HDL-C ratio and hypercalcemia had the highest odds ratio among all the risk factors (high TC/HDL-C: OR = 1.52, 95% CI 1.31–1.77, *P* <  0.001 and hypercalcemia: OR = 1.56, 95% CI 1.41–1.72, *P* <  0.001).

**Table 2:** Multivariable logistic regression of weight status and cardiovascular risk factors for moderately/severely impaired kidney function

	Model 1^a^		Model 2^b^	
	OR (95% CI)	*P*	OR (95% CI)	*P*
Weight status				
BMI, ref.: normal				
Overweight (*n* = 12,407)	1.32 (1.21–1.44)	< 0.001	1.25 (1.12–1.39)	< 0.001
Obese (*n* = 6852)	1.58 (1.43–1.75)	< 0.001	1.28 (1.12–1.45)	< 0.001
High waist circumference (*n* = 10,187)	1.29 (1.19–1.40)	< 0.001	1.10 (0.99–1.22)	0.052
High WHR (*n* = 15,673)	1.27 (1.17–1.37)	< 0.001	1.11 (1.00–1.23)	0.039
High body fat mass (*n* = 15,215)	1.24 (1.15–1.35)	< 0.001	1.06 (0.97–1.17)	0.219
Cardiovascular risk factors				
Hypertension (*n* = 11,779)	1.57 (1.44–1.70)	< 0.001	1.43 (1.29–1.58)	< 0.001
Diabetes (*n* = 3974)	1.48 (1.34–1.63)	< 0.001	1.30 (1.15–1.48)	< 0.001
High TG (*n* = 5627)	1.51 (1.36–1.67)	< 0.001	1.44 (1.27–1.63)	< 0.001
High TC (*n* = 7510)	1.44 (1.32–1.58)	< 0.001	1.39 (1.25–1.55)	< 0.001
Low HDL-C (*n* = 2335)	1.41 (1.22–1.64)	< 0.001	1.40 (1.18–1.67)	< 0.001
High LDL-C (*n* = 5709)	1.40 (1.27–1.54)	< 0.001	1.30 (1.16–1.46)	< 0.001
High TC/HDL-C (*n* = 3527)	1.62 (1.44–1.84)	< 0.001	1.52 (1.31–1.77)	< 0.001
Hypercalcemia (*n* = 12,589)	1.52 (1.40–1.65)	< 0.001	1.56 (1.41–1.72)	< 0.001
High phosphorus (*n* = 420)	1.10 (1.01–1.20)	0.037	1.11 (1.00–1.23)	0.046

*BMI* body mass index, *WHR* waist-to-hip ratio, *TG* triglycerides, *TC* total cholesterol, *HDL-C* high density lipoprotein-cholesterol, *LDL-C* low density lipoprotein-cholesterol, *TC/HDL-C* total cholesterol-to-HDL-C ratio

^a^Model 1 was adjusted for age and gender (for weight status category). For cardiovascular risk factors category, model 1 was adjusted for age, gender, and BMI

^b^Model 2 was adjusted for age, gender, smoking status, drinking status, physical activity, high cardiovascular risk profile, hypertension status, diabetes status, albumin, and CRP (for weight status category). For cardiovascular risk factor category, model 2 was adjusted for age, gender, BMI, smoking status, drinking status, physical activity, high cardiovascular risk profile, hypertension status (except hypertension variable), diabetes status (except diabetes variable), albumin, and CRP

### Dietary pattern in relation to weight status, cardiovascular risk factors, and the severity of impaired kidney function

The RRR-derived kidney function-related dietary pattern showed that food groups such as preserved vegetables, processed meat or fish, rice or flour products, meat, soy sauce, organ meats, fried rice or flour products, and instant noodles were positively correlated with dietary pattern scores (factor loading ≥0.20). In contrast, food groups like fruits, dark-colored vegetables, bread, and beans or bean products were negatively correlated with dietary pattern scores (factor loading ≥ − 0.20) (Table 3). The cumulative percentage of variation explained by RRR-derived kidney function-related dietary pattern was 6.67%. The eight response variables were explained 2.7% for the total variation and largely driven by the explained variation in WHR (6.7%), TC/HDL-C ratio (2.6%), and TG (2.2%). The baseline characteristics of the participants across tertiles of dietary pattern scores are shown in Additional file 2: Table S2. Participants with higher adherence to the dietary pattern were likely to be males, younger, current smokers and drinkers, inactive, heavier, hypertensive or diabetic, and with abnormal blood lipid levels.

**Table 3:** Factor loadings and variance of dietary pattern scores identified by the reduced rank regression model

Food group	Explained variance (%)	Factor loading^a^
Preserved vegetables, processed meat or fish	21.35	0.38
Rice/flour p**r**oducts	15.32	0.32
Meat	14.69	0.32
Soy sauce or other dipping sauce	14.07	0.31
Organ meats	13.79	0.31
Fried rice/flour products	8.83	0.25
Instant noodles	6.37	0.21
Fruit	8.20	−0.24
Dark-colored vegetables	7.70	−0.23
Breads	7.47	−0.23
Beans/bean products	6.92	−0.22
Eggs	5.33	0.19
Seafood	4.86	0.18
Deep fried foods	4.31	0.17
Dairy products	3.60	−0.16
Sugary drinks	1.24	0.09
Milk	0.76	−0.07
Light-colored vegetables	0.70	−0.07
Root crops	0.62	−0.07
Fried vegetables/salad dressing	0.35	−0.05
Jam/honey	0.09	0.02
Whole grains	0.08	−0.02

^a^ factor loadings are the correlations between food groups and dietary pattern scores. A positive factor loading value of food groups indicates a positive correlation with dietary pattern score, and vice versa

The kidney function-related dietary pattern scores across tertiles in relation to weight status, cardiovascular risk factors, and the severity of impaired kidney function are demonstrated in Table 4. A multivariable logistic regression analysis demonstrated that participants who showed higher adherence to the dietary pattern (tertile 2 and tertile 3 of dietary pattern scores) were strongly associated with increased overweight (tertile 2: OR = 1.13, 95% CI 1.06–1.21, tertile 3: OR = 1.36, 95% CI 1.27–1.46, *P* all < 0.001) and obesity (tertile 2: OR = 1.43, 95% CI 1.31–1.57, tertile 3: OR = 2.07, 95% CI 1.89–2.27, *P* all < 0.001) risk by 13–36% and 43–107%, respectively. Participants in tertile 3 of dietary pattern scores were also positively associated with all the cardiovascular risk factors except for hypertension (OR = 1.04, 95% CI 0.96–1.12, *P* = 0.31), low HDL-C (OR = 1.00, 95% CI 0.88–1.15, *P* = 0.96) and high serum phosphorus levels (≥ 1.2 mmol/L) (OR = 1.00, 95% CI 0.94–1.07, *P* = 0.98). Furthermore, the dietary pattern scores of tertile 2 and tertile 3 were significantly associated with a higher risks of moderately/severely impaired kidney function (tertile 2: OR = 1.12, 95% CI 1.00–1.25, *P* <  0.05, tertile 3: OR = 1.15, 95% CI 1.02–1.29, *P* <  0.05) and having BUN ≥7.14 mmol/L (tertile 2: OR = 1.31, 95% CI 1.16–1.47, *P* <  0.001, tertile 3: OR = 1.32, 95% CI 1.18–1.49, *P* <  0.001) (data not shown).

**Table 4:** The association of dietary pattern scores with weight status, cardiovascular risk factors, and the severity of impaired kidney function (*n* = 41,128)^a^

	Dietary pattern scores				
	Tertile 1 (Ref)	Tertile 2^b^		Tertile 3^c^	
		OR (95% CI)	*P*	OR (95% CI)	*P*
Weight status					
BMI					
Overweight	1.00	1.13 (1.06–1.21)	< 0.001	1.36 (1.27–1.46)	< 0.001
Obese	1.00	1.43 (1.31–1.57)	< 0.001	2.07 (1.89–2.27)	< 0.001
High waist circumference	1.00	1.28 (1.19–1.38)	< 0.001	1.72 (1.60–1.85)	< 0.001
High WHR	1.00	1.24 (1.16–1.32)	< 0.001	1.70 (1.59–1.82)	< 0.001
High body fat mass	1.00	1.24 (1.17–1.33)	< 0.001	1.58 (1.48–1.68)	< 0.001
Cardiovascular risk factors					
Hypertension	1.00	1.02 (0.95–1.10)	0.62	1.04 (0.96–1.12)	0.31
Diabetes	1.00	1.12 (1.00–1.24)	0.047	1.15 (1.03–1.28)	0.014
High TG	1.00	1.13 (1.03–1.24)	0.012	1.24 (1.13–1.37)	< 0.001
High TC	1.00	1.03 (0.95–1.11)	0.47	1.23 (1.14–1.33)	< 0.001
Low HDL-C	1.00	1.05 (0.92–1.20)	0.49	1.00 (0.88–1.15)	0.96
High LDL-C	1.00	1.01 (0.93–1.10)	0.78	1.17 (1.08–1.28)	< 0.001
High TC/HDL-C	1.00	1.14 (1.01–1.28)	0.032	1.31 (1.17–1.47)	< 0.001
Hypercalcemia	1.00	1.05 (0.98–1.12)	0.29	1.10 (1.02–1.17)	0.003
High phosphorus (≥ 1.2 mmol/L)	1.00	0.98 (0.92–1.04)	0.51	1.00 (0.94–1.07)	0.98
Severity of kidney function					
Moderately/severely impaired	1.00	1.12 (1.00–1.25)	0.049	1.15 (1.02–1.29)	0.019

*BMI* body mass index, *WHR* waist-to-hip ratio, *TG* triglycerides, *TC* total cholesterol, *HDL-C* high density lipoprotein-cholesterol, *LDL-C* low density lipoprotein-cholesterol, TC/HDL-C total cholesterol-to-HDL-C ratio

^a^Adjusted for model 2. Weight status category was adjusted for age, gender, smoking status, drinking status, physical activity, high cardiovascular risk profile, hypertension status, diabetes status, albumin, and CRP. Cardiovascular risk factors and the severity of kidney function categories were adjusted for age, gender, BMI, smoking status, drinking status, physical activity, high cardiovascular risk profile, hypertension status (except hypertension variable), diabetes status (except diabetes variable), albumin, and CRP. Tertile 1 (dietary pattern score: −1.34-1.22, *n* = 13,769) was used for the reference

^b^Tertile 2 (dietary pattern scores: 1.23–1.89, *n* = 13,656)

^c^Tertile 3 (dietary pattern scores: 1.90–6.55, *n* = 13,703)

## Discussion

To our knowledge, the present study is the first study that identify kidney function-related dietary pattern by using RRR. Overall, we found that kidney function-related dietary pattern was correlated with increased obesity risk and exacerbation of cardiovascular risk factors. In this dietary pattern, the food groups containing preserved or processed foods, meat, organ meats, and sauces contributed to 64% explained variation. Adding soy sauce or other sauces to preserve or process foods and meat is common in Taiwanese’s culture. Consistent with our study, a diet rich in meat and processed foods was associated with increased body weight in Asian and US adults [31, 32]. Meat, processed foods, and organ meats are commonly high in calories, saturated fat, and cholesterol, which may contribute to a surplus of energy intake. We also found that participants who ate preserved or processed foods, meat, organ meats, and sauces were also likely to consume rice or flour products and noodles. Similar studies conducted in Korean and Japanese population reported that diet rich in white rice was correlated with high risk of obesity [33, 34]. However, Xu and colleagues found an inverse association between the traditional Chinese dietary pattern, characterized by high intake of rice and pork, with the risk of obesity [35]. The conflicting findings might be due to the different food groups in the dietary pattern, lifestyle, and eating behavior in China. In our study, more than 70% of participants were physically inactive, which may also contribute to these different results. In addition, a recent population study in adults has reported that 1 SD increment of fruits and vegetables intake was inversely associated with BMI by 0.12 kg/m^2^, waist circumference by 0.40 cm, and percentage fat mass by 0.30% [36]. Fruits and vegetables are commonly known to have a higher amount of dietary fiber, phytochemicals, vitamins, and minerals, which may enhance satiety and lead to a lower energy absorption. In addition, fruits and vegetables also have anti-oxidative effects against obesity-induced oxidative stress [36]. However, our RRR derived kidney function-related dietary pattern was characterized by a low fruits and vegetables intake and this may increase the risk of obesity.

Our study reported that kidney function-related dietary pattern was correlated with an increased abnormality of most cardiovascular risk factors, except for hypertension, low HDL-C, and high serum phosphorus levels. The relationship between dietary pattern and hypertension or high serum phosphorus levels was weakened by potential confounders after adjusting for covariates in model 2; however, it remains positively correlated after adjusting for age, sex, and BMI (tertile 3: OR = 1.07, 95% CI 1.01–1.14, *P* <  0.05 for hypertension and OR = 1.06, 95% CI 1.01–1.12, *P* <  0.05 for high phosphorus levels). Other studies have found that dietary pattern consisting of high consumption in animal fat, processed meat, organ meats, or refined carbohydrate was positively associated with cardiovascular risk factors [11, 37, 38]. An imbalance between saturated and unsaturated fats and low fiber content in the animal food, together with high salt content in processed food, could have an influence over blood lipids, blood pressure, and blood glucose levels [38]. In comparison, a healthy dietary pattern with high intakes of whole grains, fruits, and vegetables was found to reduce TC by 0.07 mmol/L, LDL-C by 0.05 mmol/L, TG by 0.22 mmol/L, and increase HDL-C by 0.01 mmol/L in the study of multi-ethnic Asian people [9]. Another study conducted in a Korean adult population reported that an increased dietary fat intake (% of energy) was associated with increased TC and LDL-C levels but inversely associated with the risk of having low HDL-C and high TG levels [39]. In contrast, dietary carbohydrate intake (% of energy) was positively correlated with increased TG and low HDL-C levels [37, 39], but negatively correlated with increased TC and LDL-C levels [39]. Our RRR-derived kidney function-related dietary pattern was characterized by high intake of both fat and carbohydrate, and this might be the reason that there was no association between this dietary pattern and low HDL-C levels. Taken altogether, our findings suggest that kidney function-related dietary pattern may alter blood lipid profile and glucose metabolism, and thus may lead to increase cardiovascular disease risk.

In the present study, RRR-derived kidney function-related dietary pattern increased moderately/severely impaired kidney function risk by 12–15%. Similarly, recent studies also stated that red meat, processed meat, saturated fat, and refined carbohydrate were associated with rapid eGFR decline and kidney failure [12, 40, 41]. Meat, processed foods, and refined carbohydrate are foods high in dietary acid load [42]. A high dietary acid load is known to be related to the progression of CKD and end-stage renal disease [43]. On the other hand, a diet high in plant protein, fruits, and vegetables is commonly known as an alkaline diet, and this type of diet was associated with reduce renal acid load and kidney injury [44]. Our RRR-derived kidney function-related dietary pattern was low in fruits, vegetables, and beans consumption, which explained the positive association between dietary pattern and the severity of impaired kidney function.

Furthermore, overweight or obese weight status and all cardiovascular risk factors were positively correlated with moderately/severely impaired kidney function. Abdominal adiposity has been suggested to play a crucial role in exacerbating kidney disease by stimulating chronic inflammation or endocrine dysfunction [45]. In contrast, a recent study in Taiwan reported that CKD patients with overweight or obese status was not significantly associated with decline in eGFR [46]. Another study in South Korea found that either obesity or central obesity was associated with increased risk of stage 3a CKD, but not significantly correlated with increased risk of advanced stage 4/5 CKD [45]. Both studies suggested that waist circumference or central obesity might be a better predictor for the risk of obesity-related disease [45, 46]. BMI alone is insufficient to indicate central obesity due to the variations in individual’s body composition. However, our study found that BMI (*P* <  0.001), waist circumference (*P* = 0.052), and WHR (*P* = 0.039) were associated with advanced impaired kidney function. Our results also suggest that cardiovascular risk factors were correlated with increased moderately/severely impaired kidney function risk by 11–56%. Consistent with our results, hypertension, diabetes, and abnormal lipid profile were independent risk factors to develop severe CKD stages [47–49]. Moreover, hypercalcemia is considered as cardiovascular risk factors because elevated serum calcium levels accelerated vascular calcification and were associated with increased mortality in CKD patients [8, 50]. Thus, hypercalcemia explained the highest odds ratio (OR = 1.56) in relation to moderately/severely impaired kidney function risk in our study.

The strength of this study is that we used RRR to derive kidney function-related dietary pattern. The RRR method, which is an advanced method to identify a diet-and-disease relationship, can generate potential mediators between dietary pattern and the disease of interest. Compared with factor analysis, patterns derived from RRR is more likely to be associated with the disease of interest because the patterns are driven by disease-specific responses [19]. The RRR model may extract dietary pattern scores by maximizing the explained variation in the biomarkers for a specific diet-related disease [51]. In comparison, PCA focuses only to explain the total variation in intake of food groups and does not provide an explanation of the variation in important biomarkers [13]. Additionally, the RRR method allows researchers to identify the percentage variation from predictor variables and response variables, which both contributing to the dietary factor. Extracted factor scores can be evaluated by their corresponding response scores and by the explained variation in predictor variables. Thus, the association between food groups and response variables can be used to interpret the beneficial effects of individual food group as components of predictor variables in the dietary pattern [13]. However, this method requires prior knowledge to select intermediate biomarkers-related disease. Selecting response variables can be personally subjective and may not completely reflect the current state of knowledge. Thus, it may result in different patterns in different studies. Meanwhile, our study has several limitations. First, the cross-sectional study design cannot establish a causal relationship and it only shows a condition of one point-in-time. Hence, the possibility of reversed causation also exists. Future studies using prospective cohort or randomized trial designs are needed to explain and confirm a causal relationship between kidney function-related dietary patterns and cardiovascular risk factors. Second, relatively lower number of the participants with moderately or severely impaired kidney function compared to those with mildly impaired kidney function. Third, a self-reported FFQ may have some reporting bias, errors, and only provides information on habitual food consumption but cannot provide accurate information on actual nutrient intake for an individual. Fourth, the clinical definition of CKD is either decreased kidney function (eGFR < 60 mL/min/1.73 m^2^) in the absence of persistent albuminuria or having kidney damage (albumin-creatinine ratio > 30 mg/g), which presents for 3 months or more. Although, MJ Health Institute provided periodic health check-up (on average one examination per year per person) for its members, but not all participants had the annual examination and the clinical diagnosis of CKD cannot be made based on single measurement only. Therefore, the results found in this study may not truly represent the clinically diagnosed CKD participants. Finally, we have adjusted our results with some potential confounders. However, there are still some confounders that should be considered in the future study such as energy and protein intake and the use of renal medications, which can influence the findings of the present study.

## Conclusion

In conclusion, our findings suggest that kidney function-related dietary pattern with high intake of preserved or processed foods, meat, organ meats, rice/flour products, and instant noodles but low intake of fruit, vegetables, bread, and beans was positively correlated with abnormal weight status and cardiovascular risk factors. This type of dietary pattern may further increase the risk of cardiovascular disease and the severity of impaired kidney function.

## Additional files

Additional file 1: **Table S1.** Adjusted Spearman’s correlation coefficient (*r*) between the variables and estimated glomerular filtration rate ^a^. (DOCX 24 kb)

Additional file 2: **Table S2.** Baseline characteristics of participants across tertiles of dietary pattern scores ^a^. (DOCX 19 kb)

## Abbreviations

BMI: body mass index
BUN: blood urea nitrogen
CKD: chronic kidney disease
CRP: C-reactive protein
CVD: cardiovascular disease
DBP: diastolic blood pressure
eGFR: estimated glomerular filtration rate
FBG: fasting blood glucose
HDL-C: high density lipoprotein-cholesterol
LDL-C: low density lipoprotein-cholesterol
RRR: reduced rank regression
SBP: systolic blood pressure
TC: total cholesterol
TC/HDL-C: total cholesterol-to-HDL-C ratio
TG: triglycerides
WHR: waist-to-hip ratio
