# Supplementary material for: Association of kidney function-related dietary pattern, weight status, and cardiovascular risk factors with severity of impaired kidney function in middle-aged and older adults with chronic kidney disease: a cross-sectional population study
Source: Nutr J. 2019 Apr 22;18:27. doi: 10.1186/s12937-019-0452-4 (PMC6477746; doi:10.1186/s12937-019-0452-4)
Supplement: Supplementary file 1 — Table S1. Adjusted Spearman’s correlation coefficient (r) between the variables and estimated glomerular filtration rate a. (DOCX 24 kb) [file 12937_2019_452_MOESM1_ESM.docx]

Association of kidney function-related dietary pattern, weight status, and cardiovascular risk factors with severity of impaired kidney function in middle-aged and older adults with chronic kidney disease: a cross-sectional population study

**Additional File 1**

**Table S1.** Adjusted Spearman’s correlation coefficient (*r*) between the variables and estimated glomerular filtration rate ^a^

| Variable | *r* | *P* |
| --- | --- | --- |
| WHR | -0.100 | < 0.001 |
| TG | -0.045 | < 0.001 |
| LDL-C | -0.043 | < 0.001 |
| TC/HDL-C | -0.075 | < 0.001 |
| Calcium | -0.067 | < 0.001 |
| Phosphorus | -0.064 | < 0.001 |
| BUN | -0.351 | < 0.001 |
| Creatinine | -0.720 | < 0.001 |
| WHR waist-to-hip ratio, TG triglycerides, LDL-C low density lipoprotein-cholesterol, TC/HDL-C total cholesterol-to-HDL-C ratio, BUN blood urea nitrogen.  ^a^ Adjusted for age, gender, BMI (except WHR), smoking status, drinking status, physical activity, cardiovascular status, hypertension status, diabetes status, albumin, and CRP. | | |

**Additional File 2**

**Table S2.** Baseline characteristics of participants across tertiles of dietary pattern scores ^a^

|  | T1  (*n* = 13,769) | T2  (*n* = 13,656) | T3  (*n* = 13,703) | *P* ^b^ |
| --- | --- | --- | --- | --- |
| Age (y) | 53.5 ± 9.6 | 52.7 ± 10.0 | 51.6 ± 10.0 | < 0.001 |
| Sex, males | 5,514 (40.0) | 7,049 (51.6) | 8,813 (64.3) | < 0.001 |
| Smoking status, current | 1,109 (8.1) | 2,037 (14.9) | 3,571 (26.1) | < 0.001 |
| Drinking status, yes | 1,369 (9.9) | 1,965 (14.4) | 3,086 (22.5) | < 0.001 |
| Physical activity, yes | 3,974 (28.9) | 3,530 (25.8) | 3,310 (24.2) | < 0.001 |
| Cardiovascular status | 856 (6.2) | 840 (6.2) | 793 (5.8) | 0.274 |
| Hypertension status | 3,709 (26.9) | 3,937 (28.8) | 4,133 (30.2) | < 0.001 |
| Diabetes status | 1,125 (8.2) | 1,334 (9.8) | 1,515 (11.1) | < 0.001 |
| Weight status | | | | |
| BMI (kg/m^2^) | 23.4 ± 3.1 | 24.0 ± 3.3 | 24.6 ± 3.5 | < 0.001 |
| Waist circumference | 77.4 ± 9.2 | 79.6 ± 9.5 | 82.1 ± 10.0 | < 0.001 |
| Hip circumference | 94.6 ± 5.6 | 95.2 ± 5.9 | 96.0 ± 6.2 | < 0.001 |
| WHR | 0.8 ± 1.6 | 0.8 ± 1.1 | 0.9 ± 1.4 | < 0.001 |
| Body fat mass (%) | 27.3 ± 6.6 | 27.4 ± 7.0 | 27.4 ± 7.0 | 0.859 |
| Cardiovascular risk factors | | | | |
| SBP (mmHg) | 121.5 ± 18.0 | 122.4 ± 18.3 | 123.5 ± 18.2 | < 0.001 |
| DBP (mmHg) | 72.7 ± 11.5 | 73.8 ± 11.8 | 75.1 ± 11.9 | < 0.001 |
| FBG (mmol/L) | 5.8 ± 1.2 | 5.9 ± 1.2 | 6.0 ± 1.4 | < 0.001 |
| TG (mmol/L) | 1.3 ± 0.9 | 1.4 ± 1.0 | 1.6 ± 1.3 | < 0.001 |
| TC (mmol/L) | 5.2 ± 0.9 | 5.3 ± 0.9 | 5.4 ± 0.9 | < 0.001 |
| HDL-C (mmol/L) | 1.6 ± 0.4 | 1.5 ± 0.4 | 1.4 ± 0.4 | < 0.001 |
| LDL-C (mmol/L) | 3.1 ± 0.8 | 3.1 ± 0.8 | 3.2 ± 0.8 | < 0.001 |
| TC/HDL-C | 3.5 ± 0.9 | 3.6 ± 0.9 | 3.8 ± 1.0 | < 0.001 |
| Calcium (mg/dL) | 2.3 ± 0.1 | 2.3 ± 0.1 | 2.3 ± 0.1 | < 0.001 |
| Phosphorus (mg/dL) | 1.2 ± 0.2 | 1.1 ± 0.2 | 1.1 ± 0.2 | < 0.001 |
| Albumin, inflammatory biomarker, and kidney function | | | | |
| Albumin (g/dL) | 4.4 ± 0.2 | 4.5 ± 0.2 | 4.5 ± 0.2 | < 0.001 |
| CRP (nmol/L) | 20.4 ± 42.3 | 22.4 ± 52.1 | 24.8 ± 48.8 | < 0.001 |
| BUN (mmol/L) | 5.0 ± 1.5 | 5.2 ± 1.4 | 5.3 ± 1.5 | < 0.001 |
| Creatinine (µmol/L) | 86.5 ± 24.2 | 89.5 ± 20.8 | 92.4 ± 21.1 | < 0.001 |
| eGFR (mL/min/1.73 m^2^) | 73.9 ± 9.6 | 73.7 ± 9.8 | 74.0 ± 10.0 | 0.002 |
| Proteinuria |  |  |  | < 0.001 |
| +1 | 13,302 (96.6) | 13,043 (95.5) | 12,966 (94.6) |  |
| +2 | 265 (1.9) | 347 (2.5) | 429 (3.1) |  |
| ≥ +3 | 202 (1.5) | 266 (2.0) | 313 (2.3) |  |
| BMI body mass index, WHR waist-to-hip ratio, SBP systolic blood pressure, DBP diastolic blood pressure, FBG fasting blood glucose, TG triglycerides, TC total cholesterol, HDL-C high density lipoprotein-cholesterol, LDL-C low density lipoprotein-cholesterol, TC/HDL-C total cholesterol-to-HDL-C ratio, CRP C-reactive protein, BUN blood urea nitrogen, eGFR estimated glomerular filtration rate.  ^a^ Continuous data are presented as mean ± SD, and categorical data are presented as numbers (percentage).  ^b^ The *P*-value was analyzed using Kruskal-Wallis test for continuous variables and chi-square test for categorical variables. | | | | |
